# Towards personalized medicine – the role of methotrexate

**Published:** 2011-03

**Authors:** Sita Naik

**Affiliations:** Former Professor & Head, Department of Immunology, Sanjay Gandhi Postgraduate Institute of Medical Science, Lucknow 226 014, India sitanaik@gmail.com

More than half a century ago, an observation that folic acid antagonists interfere with the normal growth of cells led to the introduction, by Farber and colleagues of methotrexate (MTX) in the treatment regimen of children with acute lymphoblastic leukaemia^1^. Soon thereafter, MTX was reported to be effective in controlling disease activity both in patients with psoriatic arthritis and rheumatoid arthritis (RA)^2^. MTX continues to be the most widely used disease-modifying antirheumatic drug (DMARD). Patients with these chronic conditions require prolonged and often life-long treatment. However, the drug is not without toxic effects, and 10 to 30 per cent of patients discontinue treatment due to side effects^3^,^4^. Also, the dose of MTX required for effective control of disease activity varies among patients and the overall response rates vary between 35-65 per cent^5^. Currently, no reliable tests are available to predict MTX efficacy or toxicity.

In this issue, Ghodke *et al*^6^ have reported single nucleotide polymorphisms (SNPs) across intracellular folate metabolic pathways in healthy Indian subjects. Folates are single carbon donors for the synthesis of purines, pyrimidines, and methionine and hence critical for DNA synthesis and cellular function. The genes they have looked at are involved in the intracellular transport (reduced folate carrier 1 or *RFC1*), and conversion (γ glutamyl hydrolase or *GGH*) of methotrxate, metabolism of purine and pyrimidine (methylenetetrahydrofolate reductase or *MTHFR*, thymidylate synthase or *TS*, methionine synthase or MS, serine hydroxymethyltransferase I or *SHMT 1*, aminoimidazol carboxamide ribinucleotide transformylase or *ATIC*, methionine synthase reductase or *MTRR*) and efflux of the drug (multidrug resistance protein 1 or MDR1). While MTX inhibits several of the folate-dependent enzymes, such as DHFR, ATIC, and TS resulting in cellular folate depletion, genetic variants in any of these genes can affect enzyme efficacy, and in turn, influence intracellular folate levels. What is the significance of the data reported by Ghatge *et al*^6^?

The study of inherited predisposition and metabolic differences that influence inter-individual variability of responses to drugs is a rapidly growing field^7^,^8^. Pharmacogenetics, as this is termed, studies the genetic polymorphisms that correspond to sequence variations in a gene, which are single base changes that can occur at any site in the DNA molecule, in the 5’-regulatory sequence (promoter), the coding region or the untranslated 3’-region after the coding sequence. Single base changes that arise at a frequency of >1 per cent, are called single nucleotide polymorphisms (SNPs) and these may have marked impact on a patient’s response to a particular agent. However, therapeutic response is not dependent on a unique gene but on multiple genes interacting with one another^9^.

Due to the individual variability in both response and propensity to have side effects, there has been considerable interest in trying to predict response as well as toxicity of MTX in a given patient. While it has not been possible to find any clear-cut relationship between demographic/clinical factors such as age, sex, duration or severity of diseases or between laboratory parameters such as c0 reactive protein (CRP) and rheumatoid factor (RF) levels and RA response to MTX, there has been better success with genetic markers^10^. The most widely reported of these have been with MTX toxicity and C677T and A1298C polymorphisms in *MTHFR* gene^11^. Other reports have also implicated a number of other SNPs in genes of the folate metabolism pathway, including *MDR1, ABCB1, RFC, ATIC,* TS enhance region (TSER), adenosine monophosphate demaniase (*AMPDA*) and inosine triphosphat pyrophosphate (*ITPA*)^12^–^16^. Many of these reported associations are not very strong and it would be logical to assume that since MTX acts on several metabolic pathways in a polygenic disease like RA, a single genetic locus would probably be unable to adequately predict the response. In addition, the associations are also compounded by the variable penetrance of different variants. Wessels *et al*^17^ established a model for predicting MTX efficacy in RA patients which consisted of sex, rheumatoid factor and smoking status, the DAS, and 4 polymorphisms in the *AMPD1, ATIC, ITPA,* and *MTHFD1* genes. This prediction model was transformed into a scoring system ranging from 0 to 11.5. Scores of <3.5 had a true positive response rate of 95 per cent. Scores of >6 had a true negative response rate of 86 per cent. Sixty per cent of the patients were categorized as either responders or non responders^17^.

Since folic acid supplementation can reduce the toxicity of MTX, the frequency of side effects is confounded by the concurrent administration of folic acid as well as the variability in the dosage in various studies^18^. Hence, the real predictive power of these markers has not been appropriately examined in many studies and may explain the lack of consistency in the associations in all studies^19^,^20^. Also, inadequate sample size diminishes the power of a study. The frequency of gene polymorphisms shows considerable variation between populations^21^. Appropriate sample size calculation is dependent on the frequencies in the normal population and hence it is important to establish normal values for the Indian population.

Role of genes not involved in folate metabolism, but related to the disease pathogenesis such as major histocompatibility genes and Interleukin (IL)-1, IL-1 receptor antagonist has also been evaluated. It is clear that the way forward will be to simultaneously examine multiple genes, taking forward the rationale used by Wessels *et al*^17^. The advances in technology now allow us to assess several thousand genes simultaneously using microarrays. Microarrays have been used to investigate various aspects of RA^22^,^23^. Today robust platforms allow us to study SNPs across the whole genome. Such studies have the advantage that predictive factors can be looked for without any *a priori* bias.

The 21^st^ century post-genomic era is seeing a transition from the traditional practice of medicine which was based on the best option for a patient taken on the basis of evidence gathered from the larger group, to personalized treatment based on the individual’s response to a drug. The rapid expansion of pharmacogenomics data (*http://www.nigms.nih.gov/Initiatives/PGRN*, *http://www.pharmgkb.org/*) is being propelled by the demands of the user community. In fact, the benefits of deploying such a strategy is already being evaluated in some countries^24^. In India, we have only recently started to think in these directions and there are a few publications on genetic basis of MTX efficacy in Indian patients^20^,^25^. Establishing normal values is the first step in this direction. The study by Ghotge *et al*^6^ should be a stimulus for Indian scientists to plan robust studies for Indian patients so that they too can benefit from these advances.

## References

[1] Farber S, Diamond LK, Mercer RD, Sylvesterw RF, Wolff IA (1948). Temporary remissions in acute leukemia in children produced by folic acid antagonist, 4-aminopteroyl-glutamic acid (aminopterin). *N Engl J Med*.

[2] Gubner R, August S, Ginsberg V (1951). Therapeutic suppression of tissue reactivity. II. Effect of aminopterin in rheumatoid arthritis and psoriasis. *Am J Med Sci*.

[3] Kremer JM, Lee JK (1986). The safety and efficacy of the use of methotrexate in long-term therapy for rheumatoid arthritis. *Arthritis Rheum*.

[4] Alarcon GS, Tracy IC, Blackburn WD (1989). Methotrexate in rheumatoid arthritis. Toxic effects as the major factor in limiting long-term treatment. *Arthritis Rheum*.

[5] Bathon JM, Martin RW, Fleischmann RM, Tesser JR, Schiff MH, Keystone EC (2000). A comparison of etanercept and methotrexate in patients with early rheumatoid arthritis. *N Engl J Med*.

[6] Ghodke Y, Chopra A, Shintre P, Puranik A, Joshi K, Patwardhan B (2011). .Profiling single nucleotide polymorphisms (SNPs) across intracellular folate metabolic pathway in healthy Indians. *Indian J Med Res*.

[7] Evans WE, Relling MV (1999). Pharmacogenomics : translating functional genomics into rational therapeutics. *Science*.

[8] Ranganathan P (2005). Pharmacogenomics of tumor necrosis factor antagonists in rheumatoid arthritis. *Pharmacogenomics*.

[9] Bridges SL (2004). Genetic markers of treatment response in rheumatoid arthritis. *Arthritis Rheum*.

[10] Bansard C, Lequerreé T, Daveau1 M, Boyer O, Tron F, Salier J (2009). Can rheumatoid arthritis responsiveness to methotrexate and biologics be predicted?. *Rheumatology*.

[11] Lee YH, Song GG (2010). Association between the C677T and A1298C polymorphisms of *MTHFR* and the efficacy and toxicity of methotrexate in rheumatoid arthritis : a metanalysis. *Clin Drug Invest*.

[12] Ulrich CM, Robien K, Sparks R (2002). Pharmacogenetics and folate metabolism : a promising direction. *Pharmacogenomics*.

[13] Dervieux T, Lein DO, Park G, Barham R, Smith K, Walsh M (2003). Single nucleotide polymorphisms (SNPs) in the folate/purine synthesis pathway predict methotrexate’s effects in rhematoid arthritis. *Arthritis Rheum*.

[14] Pawlik A, Wrzesniewska J, Fiedorowicz-Fabrycy I, Gawronska-Szklarz B (2004). The MDR1 3435 polymorphism in patients with rheumatoid arthritis. *Int J Clin Pharmacol Ther*.

[15] Takatori R, Takahashi KA, Tokunaga D, Hojo T, Fujioka M, Asano T (2006). ABCB1 C3435T polymorphism influences methotrexate sensitivity in rheumatoid arthritis patients. *Clin Exp Rheumatol*.

[16] Dervieux T, Furst D, Lein DO, Capps R, Smith K, Caldwell J (2005). Pharmacogenetic and metabolite measurements are associated with clinical status in patients with rheumatoid arthritis treated with methotrexate: results of a multicentred cross sectional observational study. *Ann Rheum Dis*.

[17] Wessels JA, van der Kooij SM, le Cessie S, Kievit N, Barrera P, Allart CF (2007). A clinical pharmacogenetic model to predict the efficacy of methotrexate monotherapy in recent-onset rheumatoid arthritis. *Arthritis Rheum*.

[18] Van Ede AE, Laan RF, Rood MJ, Huizinga TW, van de Laar MA, van Denderen CJ (2001). Effect of folic or folinic acid supplementation on the toxicity and efficacy of methotrexate in rheumatoid arthritis: a forty-eight–week, multicenter, randomized, doubleblind, placebo-controlled study. *Arthritis Rheum*.

[19] Kumagai K, Hiyama K, Oyama T, Maeda H, Kohno N (2003). Polymorphisms in the thymidylate synthase and methylenetetrahydrofolate reductase genes and sensitivity to the low-dose methotrexate therapy in patients with rheumatoid arthritis. *Int J Mol Med*.

[20] Aggarwal P, Naik S, Mishra KP, Aggarwal A, Misra R (2006). Correlation between methotrexate efficacy and toxicity with C677T polymorphism of the methylenetetrahydrofolate gene in rheumatoid arthritis patients on folate supplementation. *Indian J Med Res*.

[21] Hughes LB, Beasley TM, Patel H, Tiwari HK, Morgan SL, Baggott JE (2006). Racial or ethnic differences in allele frequencies of single-nucleotide polymorphisms in the methyltetrahydrofolate reductase gene and their influence on response to methotrexate in rheumatoid arthritis. *Ann Rheum Dis*.

[22] Jarvis JN (2005). Diagnostic and prognostic potential of gene microarrays in rheumatoid arthritis. *Expert Rev Mol Diagn*.

[23] van Baarsen LG, Vijbrandts CA, Timmer TC, van der Pouw Kraan TC, Tak PP, Verweij CL (2010). Synovial tissue heterogeneity in rheumatoid arthritis in relation to disease activity and biomarkers in peripheral blood. *Arthritis Rheum*.

[24] Kim SK, Jun JB, El-Sohemy A, Bae SC (2006). Cost-effectiveness analysis of MTHFR polymorphism screening by polymerase chain reaction in Korean patients with rheumatic arthritis receiving methotrexate. *J Rheumatol*.

[25] Sharma S, Das M, Kumar A, Marwaha V, Shankar S, Aneja R (2008). Interaction of genes from influx-metabolism-efflux pathay and their influence on methotrexate efficacy in rheumatoid arthritis patients among Indians. *Pharmacogenet Genomics*.

